# (2*Z*)-2-Benzyl­idene-4-(prop-2-yn-1-yl)-2*H*-1,4-benzo­thia­zin-3(4*H*)-one

**DOI:** 10.1107/S1600536814009179

**Published:** 2014-04-26

**Authors:** Nada Kheira Sebbar, Abdelfettah Zerzouf, El Mokhtar Essassi, Mohamed Saadi, Lahcen El Ammari

**Affiliations:** a Laboratoire de Chimie Organique Hétérocyclique URAC 21, Pôle de Compétence Pharmacochimie, Av. Ibn Battouta, BP 1014, Faculté des Sciences, Université Mohammed V-Agdal, Rabat, Morocco; b Laboratoire de Chimie Organique et Etudes Physicochimiques, ENS Takaddoum, Rabat, Morocco; c Laboratoire de Chimie du Solide Appliquée, Faculté des Sciences, Université Mohammed V-Agdal, Avenue Ibn Battouta, BP 1014, Rabat, Morocco

## Abstract

The mol­ecule of the title compound, C_18_H_13_NOS, is build up from two fused six-membered rings, with the heterocyclic component linked to a benzyl­idene group and to a prop-2-yn-1-yl chain. The six-membered heterocycle adopts a distorted screw-boat conformation. The prop-2-yn-1-yl chain is almost perpendicular to the mean plane through benzo­thia­zine as indicated by the C—N—C—C torsion angle of 86.5 (2)°. The dihedral angle between the benzene rings is 47.53 (12)°. There are no specific inter­molecular inter­actions in the crystal packing.

## Related literature   

For the pharmacological activity of benzo­thia­zine derivatives, see: Aotsuka *et al.* (1994 ▶); Fujimura *et al.* (1996 ▶); Rathore & Kumar (2006 ▶); Fringuelli *et al.* (1998 ▶). For related structures, see: Sebbar *et al.* (2014*a* ▶,**b* ▶)*; Zerzouf *et al.* (2001 ▶). For conformation analysis, see: Cremer & Pople (1975 ▶).
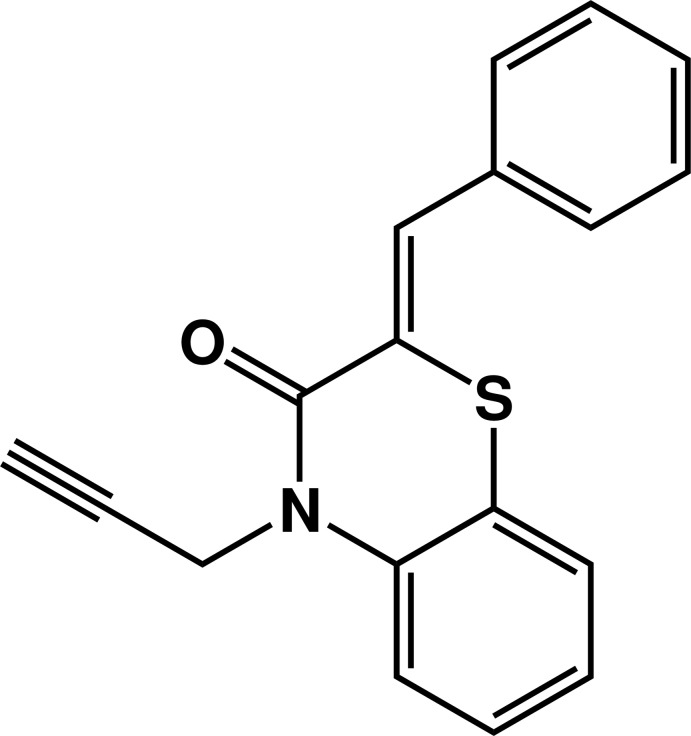


## Experimental   

### 

#### Crystal data  

C_18_H_13_NOS
*M* *_r_* = 291.35
Orthorhombic, 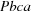

*a* = 9.0254 (13) Å
*b* = 7.7388 (12) Å
*c* = 42.488 (7) Å
*V* = 2967.6 (8) Å^3^
*Z* = 8
Mo *K*α radiation
μ = 0.22 mm^−1^
*T* = 296 K
0.42 × 0.36 × 0.31 mm

#### Data collection  

Bruker X8 APEX diffractometer
Absorption correction: multi-scan (*SADABS*; Bruker, 2009 ▶) *T*_min_ = 0.579, *T*_max_ = 0.746
14350 measured reflections
3248 independent reflections
2227 reflections with *I* > 2σ(*I*)
*R*_int_ = 0.038

#### Refinement  

*R*[*F* ^2^ > 2σ(*F* ^2^)] = 0.046
*wR*(*F* ^2^) = 0.124
*S* = 1.02
3248 reflections
190 parameters
H-atom parameters constrained
Δρ_max_ = 0.24 e Å^−3^
Δρ_min_ = −0.30 e Å^−3^

### 

Data collection: *APEX2* (Bruker, 2009 ▶); cell refinement: *SAINT-Plus* (Bruker, 2009 ▶); data reduction: *SAINT-Plus*; program(s) used to solve structure: *SHELXS97* (Sheldrick, 2008 ▶); program(s) used to refine structure: *SHELXL97* (Sheldrick, 2008 ▶); molecular graphics: *ORTEP-3 for Windows* (Farrugia, 2012 ▶); software used to prepare material for publication: *PLATON* (Spek, 2009 ▶) and *publCIF* (Westrip, 2010 ▶).

## Supplementary Material

Crystal structure: contains datablock(s) I. DOI: 10.1107/S1600536814009179/tk5310sup1.cif

Structure factors: contains datablock(s) I. DOI: 10.1107/S1600536814009179/tk5310Isup2.hkl

Supporting information file. DOI: 10.1107/S1600536814009179/tk5310Isup3.cml

CCDC reference: 999032

Additional supporting information:  crystallographic information; 3D view; checkCIF report

## Figures and Tables

**Fig. 1.**
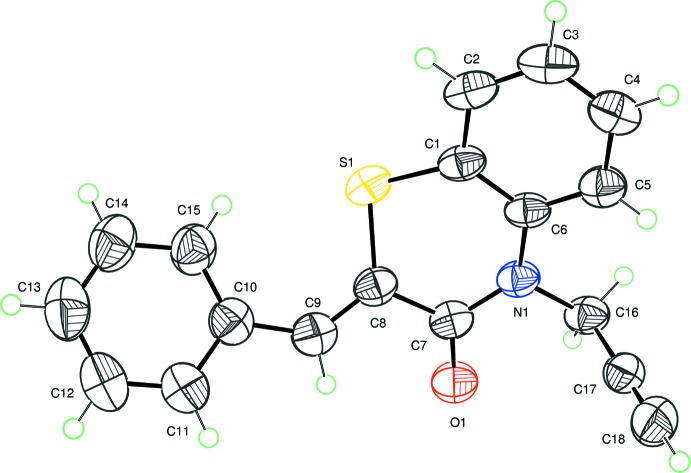
Molecular structure of the title compound with the atom-labelling scheme. Displacement ellipsoids are drawn at the 50% probability level. H atoms are represented as small circles.

## supplementary crystallographic information

### 2. Structural commentary

Benzo­thia­zine containing compounds are important due to their potential applications as treatment of diabete complications, by inhibiting aldose redu­ctase (Aotsuka *et al.*, 1994), having activities antagonists of Ca^2+^ (Fujimura *et al.*, 1996), anti­microbial and anti­fungal (Rathore & Kumar, 2006; Fringuelli *et al.*, 1998). The present work is a continuation of the investigation of the benzo­thia­zine derivatives published recently by our team (Sebbar *et al.*, 2014*a*; 2014*b*; Zerzouf *et al.*, 2001). In this work, we are inter­ested in the synthesis of the title compound for biological activities, by reacting (2*Z*)-2-(benzyl­idene)-3,4-di­hydro-2*H*-1,4- benzo­thia­zin-3-one with propargyl bromide, under phase-transfer catalysis conditions using tetra *n*-butyl ammonium bromide (TBAB) as catalyst and potassium carbonate as base (Scheme 1).

In the molecule of the title compound, the six-membered heterocycle (S1N1C1C6C7C8) of the benzo­thia­zine fragment exhibits a conformation between boat and screw boat conformation as indicated by the puckering amplitude Q = 0.6536 (17) Å, and spherical polar angle θ = 112.04 (16)°, with φ = 152.14 (18)° (Cremer & Pople, 1975). The prop-2-yn-1-yl chain is almost perpendicular to mean plane through the benzene ring, as indicated by the torsion angle C6–N1–C16–C17 of 86.5 (2)° (Fig. 1). The dihedral angle between the two planes through the benzene rings (C1 to C6 and C10 to C15) is of 47.53 (12)°.

### 5. Synthesis and crystallization

To a mixture of (2*Z*)-2-(benzyl­idene)-3,4-di­hydro-2*H*-1,4-benzo­thia­zin-3-one (0.38 g, 1.5 mmol), potassium carbonate (0.24 g, 1.8 mmol) and tetra *n*-butyl ammonium bromide (0.05 g, 0.15 mmol) in DMF (25 ml) was added propargyl bromide (0.12 ml, 1.6 mmol). Stirring was continued at room temperature for 24 h. The salt was removed by filtration and the filtrate was concentrated under reduced pressure. The residue was separated by chromatography on a column of silica gel with ethyl acetate-hexane as eluent; yellow crystals were obtained upon evaporation of the solvent (yield = 55% and m.pt = 404 K).

### 6. Refinement

The H atoms were located in a difference map and treated as riding with C—H = 0.93 Å (aromatic, acetyl­enic) and C—H = 0.97 Å (methyl­ene), and with *U*_iso_(H) = 1.2 *U*_eq_(C).

### Crystal data

**Table:**

C_18_H_13_NOS	*D*_x_ = 1.304 Mg m^−^^3^
*M**_r_* = 291.35	Melting point: 404 K
Orthorhombic, *P**b**c**a*	Mo *K*α radiation, λ = 0.71073 Å
Hall symbol: -P 2ac 2ab	Cell parameters from 3248 reflections
*a* = 9.0254 (13) Å	θ = 2.5–27.1°
*b* = 7.7388 (12) Å	µ = 0.22 mm^−^^1^
*c* = 42.488 (7) Å	*T* = 296 K
*V* = 2967.6 (8) Å^3^	Block, yellow
*Z* = 8	0.42 × 0.36 × 0.31 mm
*F*(000) = 1216	

### Data collection

**Table:**

Bruker X8 APEX diffractometer	3248 independent reflections
Radiation source: fine-focus sealed tube	2227 reflections with *I* > 2σ(*I*)
Graphite monochromator	*R*_int_ = 0.038
φ and ω scans	θ_max_ = 27.1°, θ_min_ = 2.5°
Absorption correction: multi-scan (*SADABS*; Bruker, 2009)	*h* = −10→11
*T*_min_ = 0.579, *T*_max_ = 0.746	*k* = −5→9
14350 measured reflections	*l* = −54→42

### Refinement

**Table:**

Refinement on *F*^2^	Primary atom site location: structure-invariant direct methods
Least-squares matrix: full	Secondary atom site location: difference Fourier map
*R*[*F*^2^ > 2σ(*F*^2^)] = 0.046	Hydrogen site location: inferred from neighbouring sites
*wR*(*F*^2^) = 0.124	H-atom parameters constrained
*S* = 1.02	*w* = 1/[σ^2^(*F*_o_^2^) + (0.0511*P*)^2^ + 0.8991*P*] where *P* = (*F*_o_^2^ + 2*F*_c_^2^)/3
3248 reflections	(Δ/σ)_max_ < 0.001
190 parameters	Δρ_max_ = 0.24 e Å^−^^3^
0 restraints	Δρ_min_ = −0.30 e Å^−^^3^

### Special details

**Table:**

Geometry. All e.s.d.'s (except the e.s.d. in the dihedral angle between two l.s. planes) are estimated using the full covariance matrix. The cell e.s.d.'s are taken into account individually in the estimation of e.s.d.'s in distances, angles and torsion angles; correlations between e.s.d.'s in cell parameters are only used when they are defined by crystal symmetry. An approximate (isotropic) treatment of cell e.s.d.'s is used for estimating e.s.d.'s involving l.s. planes.
Refinement. Refinement of *F*^2^ against all reflections. The weighted *R*-factor *wR* and goodness of fit *S* are based on *F*^2^, conventional *R*-factors *R* are based on *F*, with *F* set to zero for negative *F*^2^. The threshold expression of *F*^2^ > σ(*F*^2^) is used only for calculating *R*-factors(gt) *etc*. and is not relevant to the choice of reflections for refinement. *R*-factors based on *F*^2^ are statistically about twice as large as those based on *F*, and *R*- factors based on all data will be even larger.

### Fractional atomic coordinates and isotropic or equivalent isotropic displacement parameters (Å^2^)

**Table:**

	*x*	*y*	*z*	*U*_iso_*/*U*_eq_	
C1	0.2609 (2)	0.8787 (2)	0.10840 (5)	0.0479 (5)	
C2	0.1083 (2)	0.8810 (3)	0.10322 (6)	0.0565 (6)	
H2	0.0443	0.8448	0.1191	0.068*	
C3	0.0518 (2)	0.9359 (3)	0.07502 (6)	0.0615 (6)	
H3	−0.0501	0.9412	0.0720	0.074*	
C4	0.1463 (2)	0.9829 (3)	0.05121 (6)	0.0606 (6)	
H4	0.1081	1.0198	0.0320	0.073*	
C5	0.2986 (2)	0.9758 (3)	0.05563 (5)	0.0531 (5)	
H5	0.3618	1.0037	0.0391	0.064*	
C6	0.35728 (19)	0.9276 (2)	0.08436 (5)	0.0438 (5)	
C7	0.5877 (2)	0.8372 (3)	0.11202 (5)	0.0476 (5)	
C8	0.5034 (2)	0.7547 (3)	0.13808 (5)	0.0479 (5)	
C9	0.5746 (2)	0.6372 (3)	0.15553 (5)	0.0552 (5)	
H9	0.6729	0.6203	0.1498	0.066*	
C10	0.5260 (2)	0.5307 (3)	0.18189 (5)	0.0570 (5)	
C11	0.6072 (3)	0.3823 (4)	0.18816 (7)	0.0896 (9)	
H11	0.6905	0.3577	0.1761	0.107*	
C12	0.5674 (4)	0.2717 (5)	0.21174 (8)	0.1084 (11)	
H12	0.6237	0.1730	0.2154	0.130*	
C13	0.4463 (3)	0.3036 (4)	0.23001 (7)	0.0863 (8)	
H13	0.4196	0.2274	0.2460	0.104*	
C14	0.3653 (3)	0.4485 (4)	0.22456 (6)	0.0762 (7)	
H14	0.2826	0.4716	0.2369	0.091*	
C15	0.4043 (3)	0.5618 (3)	0.20089 (5)	0.0671 (6)	
H15	0.3478	0.6608	0.1977	0.081*	
C16	0.6078 (2)	1.0153 (3)	0.06611 (5)	0.0508 (5)	
H16A	0.5557	1.1143	0.0575	0.061*	
H16B	0.6966	1.0574	0.0764	0.061*	
C17	0.6504 (2)	0.9004 (3)	0.04036 (5)	0.0532 (5)	
C18	0.6844 (3)	0.8080 (4)	0.01999 (6)	0.0761 (7)	
H18	0.7116	0.7341	0.0037	0.091*	
N1	0.51305 (15)	0.9304 (2)	0.08949 (4)	0.0455 (4)	
O1	0.72273 (14)	0.8256 (2)	0.11070 (4)	0.0649 (4)	
S1	0.32280 (6)	0.82420 (9)	0.145920 (14)	0.0667 (2)	

### Atomic displacement parameters (Å^2^)

**Table:**

	*U*^11^	*U*^22^	*U*^33^	*U*^12^	*U*^13^	*U*^23^
C1	0.0428 (9)	0.0399 (10)	0.0610 (13)	0.0036 (8)	0.0089 (9)	−0.0108 (9)
C2	0.0426 (10)	0.0505 (12)	0.0763 (16)	0.0000 (9)	0.0150 (11)	−0.0098 (11)
C3	0.0394 (10)	0.0539 (12)	0.0912 (18)	0.0034 (9)	−0.0007 (11)	−0.0134 (13)
C4	0.0519 (11)	0.0600 (13)	0.0698 (15)	0.0084 (10)	−0.0087 (11)	−0.0048 (12)
C5	0.0457 (10)	0.0527 (12)	0.0610 (14)	−0.0002 (9)	0.0028 (10)	−0.0015 (10)
C6	0.0371 (9)	0.0355 (9)	0.0586 (13)	0.0003 (7)	0.0039 (8)	−0.0079 (9)
C7	0.0424 (10)	0.0479 (11)	0.0525 (12)	−0.0026 (8)	0.0020 (8)	−0.0078 (10)
C8	0.0468 (10)	0.0494 (11)	0.0476 (11)	−0.0006 (9)	0.0022 (9)	−0.0093 (10)
C9	0.0527 (11)	0.0587 (13)	0.0543 (13)	−0.0017 (10)	−0.0018 (10)	−0.0070 (11)
C10	0.0606 (12)	0.0624 (14)	0.0481 (12)	−0.0023 (10)	−0.0088 (10)	−0.0051 (11)
C11	0.0892 (18)	0.100 (2)	0.0790 (19)	0.0273 (16)	0.0084 (15)	0.0242 (17)
C12	0.117 (3)	0.110 (2)	0.098 (2)	0.032 (2)	0.006 (2)	0.045 (2)
C13	0.099 (2)	0.094 (2)	0.0654 (18)	−0.0107 (17)	−0.0083 (16)	0.0213 (16)
C14	0.0838 (16)	0.094 (2)	0.0507 (14)	−0.0108 (15)	0.0001 (13)	−0.0006 (14)
C15	0.0807 (15)	0.0700 (15)	0.0506 (14)	0.0014 (13)	0.0015 (12)	−0.0025 (12)
C16	0.0395 (9)	0.0467 (11)	0.0662 (14)	−0.0037 (8)	0.0062 (9)	0.0011 (10)
C17	0.0492 (11)	0.0534 (12)	0.0569 (14)	0.0037 (9)	0.0078 (10)	0.0095 (11)
C18	0.0952 (18)	0.0717 (17)	0.0614 (16)	0.0160 (14)	0.0134 (14)	0.0044 (14)
N1	0.0373 (7)	0.0437 (9)	0.0555 (10)	−0.0022 (6)	0.0045 (7)	−0.0020 (8)
O1	0.0399 (7)	0.0831 (11)	0.0718 (10)	−0.0003 (7)	0.0004 (7)	0.0077 (9)
S1	0.0603 (3)	0.0835 (4)	0.0565 (4)	0.0174 (3)	0.0176 (3)	0.0016 (3)

### Geometric parameters (Å, º)

**Table:**

C1—C6	1.394 (3)	C9—H9	0.9300
C1—C2	1.394 (3)	C10—C15	1.384 (3)
C1—S1	1.741 (2)	C10—C11	1.388 (3)
C2—C3	1.369 (3)	C11—C12	1.366 (4)
C2—H2	0.9300	C11—H11	0.9300
C3—C4	1.372 (3)	C12—C13	1.363 (4)
C3—H3	0.9300	C12—H12	0.9300
C4—C5	1.389 (3)	C13—C14	1.358 (4)
C4—H4	0.9300	C13—H13	0.9300
C5—C6	1.382 (3)	C14—C15	1.380 (3)
C5—H5	0.9300	C14—H14	0.9300
C6—N1	1.423 (2)	C15—H15	0.9300
C7—O1	1.224 (2)	C16—C17	1.461 (3)
C7—N1	1.375 (2)	C16—N1	1.466 (2)
C7—C8	1.488 (3)	C16—H16A	0.9700
C8—C9	1.338 (3)	C16—H16B	0.9700
C8—S1	1.7482 (19)	C17—C18	1.164 (3)
C9—C10	1.458 (3)	C18—H18	0.9300
			
C6—C1—C2	119.8 (2)	C11—C10—C9	117.1 (2)
C6—C1—S1	122.42 (15)	C12—C11—C10	121.3 (3)
C2—C1—S1	117.68 (16)	C12—C11—H11	119.3
C3—C2—C1	120.6 (2)	C10—C11—H11	119.3
C3—C2—H2	119.7	C13—C12—C11	121.0 (3)
C1—C2—H2	119.7	C13—C12—H12	119.5
C2—C3—C4	119.73 (19)	C11—C12—H12	119.5
C2—C3—H3	120.1	C14—C13—C12	118.9 (3)
C4—C3—H3	120.1	C14—C13—H13	120.5
C3—C4—C5	120.3 (2)	C12—C13—H13	120.5
C3—C4—H4	119.8	C13—C14—C15	120.8 (3)
C5—C4—H4	119.8	C13—C14—H14	119.6
C6—C5—C4	120.6 (2)	C15—C14—H14	119.6
C6—C5—H5	119.7	C14—C15—C10	121.1 (2)
C4—C5—H5	119.7	C14—C15—H15	119.4
C5—C6—C1	118.78 (17)	C10—C15—H15	119.4
C5—C6—N1	120.64 (17)	C17—C16—N1	112.83 (16)
C1—C6—N1	120.57 (18)	C17—C16—H16A	109.0
O1—C7—N1	119.64 (18)	N1—C16—H16A	109.0
O1—C7—C8	120.80 (19)	C17—C16—H16B	109.0
N1—C7—C8	119.54 (16)	N1—C16—H16B	109.0
C9—C8—C7	117.30 (18)	H16A—C16—H16B	107.8
C9—C8—S1	123.48 (16)	C18—C17—C16	179.5 (2)
C7—C8—S1	119.10 (15)	C17—C18—H18	180.0
C8—C9—C10	131.7 (2)	C7—N1—C6	125.65 (16)
C8—C9—H9	114.1	C7—N1—C16	114.90 (15)
C10—C9—H9	114.1	C6—N1—C16	118.65 (16)
C15—C10—C11	116.8 (2)	C1—S1—C8	101.50 (9)
C15—C10—C9	126.0 (2)		
